# Integrating social welfare and conservation: Improvements in multidimensional poverty index outcomes from ecological resettlements in Nepal

**DOI:** 10.1016/j.isci.2026.115580

**Published:** 2026-04-03

**Authors:** Hari Prasad Pandey, Armando Apan, Tek Narayan Maraseni

**Affiliations:** 1 University of Southern Queensland, Toowoomba, QLD 4350, Australia; 2 Department of Forests and Soil Conservation, Ministry of Forests and Environment, Kathmandu 44600, Nepal; 3 Institute of Environmental Science and Meteorology, University of the Philippines Diliman, Quezon City 1101, Philippines; 4 Northwest Institute of Eco-Environment and Resources, Chinese Academy of Sciences, Lanzhou 730000, China

**Keywords:** Natural resources, Economics, Interdisciplinary application studies

## Abstract

Ecological resettlement (ER) is widely debated and frequently linked to impoverishment, yet quantitative, multi-scale evidence remains limited. This study evaluates long-term poverty dynamics among communities resettled from Chitwan National Park and Parsa National Park in Nepal. Using data from 240 household surveys (25% sampling), five focus groups, 25 key informant interviews, and secondary sources, we compare poverty levels before resettlement, within five years after relocation, and in 2024. Multidimensional poverty index (MPI) analysis and Chi-square tests reveal a significant decline in MPI from 47.36% to 6.87%, aligning with national rural averages. Poverty incidence and intensity decreased significantly (*p* < 0.05) across both sites. MPI outcomes were strongly associated with location, ethnicity, land and livestock ownership, and access to markets, forests, schools, and healthcare. Results indicate that a well-designed ER can reduce long-term poverty while advancing conservation. Integrating income and non-income indicators, including local wealth-ranking systems, strengthens poverty assessment and informs socially inclusive conservation policy.

## Highlights

• Poverty reduction has been central to policy since the Earth summit and global goals
• MPI declines post-settlement but omits livelihood loss, cultural, and psychological disruption
• Planned resettlements can align conservation and social welfare for a win-win outcome
• Expanding MPI with socioeconomic factors can improve poverty and development tracking

## Graphical abstract

**Figure:**
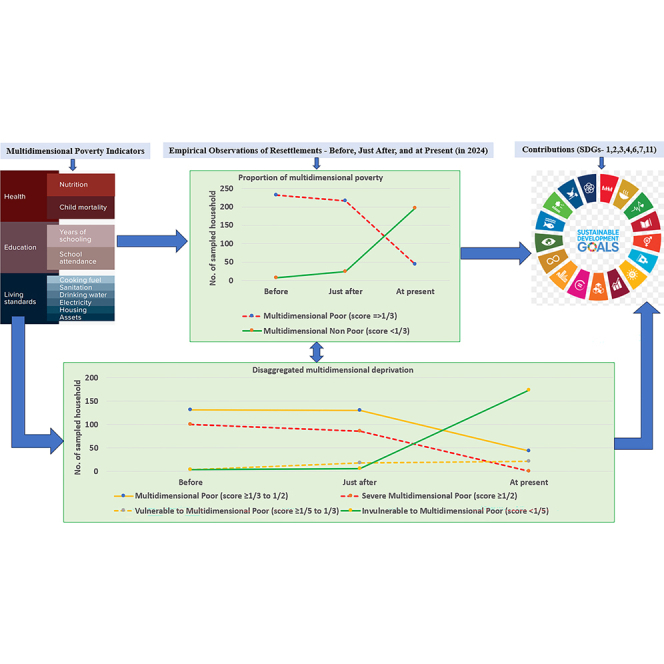

## Introduction

Balancing conservation with social needs is often challenging in the Anthropocene—an era of human dominance over climate and the environment. Conservation strategies are often criticized for neglecting social needs, making them a contentious issue in modern environmental discourse. While these efforts aim to protect natural resources and biodiversity, they frequently displace communities from their ancestral lands,^1^^,^^2^^,^^3^ disrupting traditional livelihoods and limiting access to essential services.^4^^,^^5^ A holistic evaluation can pinpoint the most affected areas, offering crucial insights into designing conservation policies that minimize negative impacts while supporting displaced populations.^6^^,^^7^^,^^8^ In the meantime, global poverty alleviation has been a priority for decades, yet conservation remains an urgent necessity.^9^^,^^10^ Notably, the world’s poorest populations often reside near biodiversity hotspots,^11^ and are disproportionately displaced for conservation purposes.^4^^,^^12^ Critics argue that conservation strategies exacerbate poverty among these vulnerable communities.^13^^,^^14^^,^^15^ However, limited studies rigorously assess the poverty levels of displaced populations, making it difficult to determine whether conservation efforts inherently impoverish communities or if they can be designed to balance ecological and social needs.

Several approaches are used to evaluate the social status of the people, such as the poverty level. Because of the robustness of a consistent account of the overall change in multidimensional poverty across time and space, the multidimensional poverty index (MPI), is considered one of the well-accepted tools to assess the poverty status at a local, regional, and global level beyond non-income dimensions.^16^^,^^17^^,^^18^ The MPI provides a nuanced understanding of poverty beyond income measures in its ability to highlight non-monetary deprivations, which include deprivations across multiple dimensions, including health, education, and living standards.^19^ The MPI is closely aligned with global development agendas, including the millennium development goals (MDGs) and the sustainable development goals (SDGs). Established in 2000, the MDGs focused on reducing extreme poverty and improving health, education, and environmental sustainability by 2015.^10^ While they primarily relied on specific indicators such as child mortality and maternal health, the MPI offers a broader framework by measuring poverty across multiple deprivations.^17^^,^^20^ Building on the MDGs, the SDGs, adopted in 2015, provide a more interconnected approach to sustainable development, with 17 goals aimed at ending poverty, protecting the planet, and ensuring prosperity for all by 2030.^9^ Poverty alleviation aligns particularly with SDG 1, which seeks to eradicate poverty in all its forms, by capturing the complexity of poverty based on cost of basic need (CBN) assessments^21^^,^^22^ but MPI covers a range of goals such as goals 2, 3, 4, 6, 7, 9, 11, 12, 13, 15, and 16, and measures poverty beyond the income-based traditional approach of CBN. By evaluating deprivations in health, education, and living standards, the MPI helps track progress toward these goals and informs policies that address poverty’s root causes, promoting sustainable and inclusive development.^17^ By leveraging the MPI, this research provides a nuanced analysis of how resettlement impacts poverty beyond mere income measures.^23^^,^^24^

Past studies have explored various aspects of resettled and displaced communities due to ecological conservation. Resettlement impacts are closely linked to forms of displacement that extend beyond voluntary resettlement, differing in spatial proximity (near versus distant), duration (temporary versus permanent), and degree of coercion (forced versus voluntary), consistent with the World Bank’s resettlement classification.^1^^,^^25^^,^^26^ Most of the previous scholarly works focus on the social dimension, highlighting how displacement leads to impoverishment—whether by restricting nature-based livelihoods, causing land-based cultural erosion, affecting identity, or a combination of these factors.^1^^,^^2^^,^^3^^,^^4^^,^^5^^,^^6^^,^^7^^,^^8^^,^^14^^,^^15^^,^^27^^,^^28^^,^^29^^,^^30^^,^^31^ In regard to the application of MPI, previous studies have largely applied the MPI at the national level using census data of over 100 developing countries^17^^,^^19^ and assessed the MPI using health and demographic data from Armenia, India, and Ethiopia,^32^ and in China.^20^ Despite growing attention to conservation-induced displacement and multidimensional poverty, several critical gaps remain. Limited research has explored temporal changes in poverty post-resettlement, gender-specific impacts, and the role of institutional support in shaping outcomes. Moreover, there is a lack of comparative analysis within displaced groups and minimal attention to spatial inequalities within resettled communities. These gaps highlight the need for a more holistic and context-specific evaluation framework.^25^^,^^33^

Furthermore, it is also important to assess whether other factors beyond the 10 MPI dimensions—such as village location, distance to services, land and livestock ownership, and household characteristics—influence poverty and strengthen the method’s credibility through empirical evidence.^16^^,^^20^^,^^32^ Moreover, research questions such as: Is it possible to achieve poverty alleviation and conservation outcomes in a win-win scenario? How do social factors contribute to poverty alleviation? What factors should be considered to address the multidimensional poverty of resettled communities? Are the existing MPI dimensions sufficient to reflect household-level deprivation? What additional factors influence human development and household deprivation? What is the conservation linkage with poverty in the Global South? remains unanswered.^25^^,^^34^^,^^35^^,^^36^ Further, as the global vision to combat poverty and Nepal’s long-term vision for 2043 is to end absolute poverty and limit multidimensional poverty to 3% by then,^21^ it is crucial to assess the MPI at its subnational level. This helps to track progress as well as to contribute to national targets. Also, a deep study in this area provides a lesson learning for assessing MPI in other countries, not limited to conservation-led impact on poverty reduction, but to other types of displacement, and at (sub)national as well.^17^^,^^25^^,^^35^^,^^37^^,^^38^ Therefore, we address these gaps in multi-scale and quantitative analysis by examining multiple factors and indicators influencing conservation and social poverty alleviation, using both qualitative and quantitative data from the globally recognized Terai Arc landscape (TAL) in Nepal (Table 1).

**Table 1:** Sample size considered for this study in the resettled villages and households (HH) across the TAL area of Nepal

National Park	Resettled villages	Sampled villages	Names of villages	Total HH in sampled villages	Sample size (HH)	Resettlement period
Parsa NP	4	4	Ramauli	228	62	2009–2013
			Pratappur	149	40	
			Rambhauri and Bhata (Krishnanagar)	96^a^	30^a^	
Chitwan NP	1	1	Padampur	516	108	1995–1998
Total	5	5	–	989	240	–

a The resettled community was relocated to a single site to ensure that residents from two evacuated villages remained together for data collection and analysis. After relocation, the site was renamed from “Rambhauribhata” to “Krishnanagar”. The timeline periods considered in this study, unless otherwise stated, are before resettlement, shortly after resettlement (first five years), and at present (survey year, 2024).

**Figure 1:**
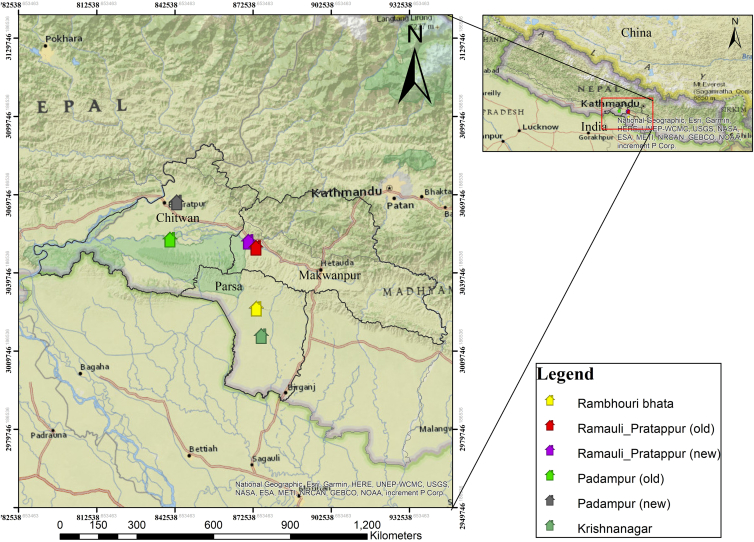
Map of the study areas showing the original residences (old sites) and the post-resettlement villages (new sites) Krishnanagar was renamed Rambhouribhata after resettlement to a new location.

In this context, our study examines multidimensional poverty in conservation-led resettlement communities to guide policies that align conservation with poverty reduction. Using the MPI (Table 2), we aim to analyze poverty dynamics before (prior to 1995 for Chitwan National Park and prior to 2009 for Parsa National Park), shortly after (within five years of those respective dates), and in 2024 (survey year). Additionally, we tested 20 other variables beyond MPI dimensions (Table 3) to determine if they have any association influencing household poverty in the study area (Figure 1). Further, we explored potential improvements to its dimensions, focusing on all five resettlement villages from two national parks in Nepal. As representative sites for global conservation and resettlement programs, our findings provide insights into conservation planning that prioritizes social well-being, enhances MPI credibility, and supports poverty tracking in alignment with the human development index, SDGs, and beyond. Specifically, this study is unique in three ways. First, the MPI applies at the subnational level to assess poverty among conservation-induced resettled communities, an approach rarely used in existing literature. Second, it expands the analysis by integrating additional socio-spatial and institutional variables beyond standard MPI dimensions to capture underlying drivers of deprivation. Third, it empirically examines the conservation-poverty nexus using mixed methods in a globally significant biodiversity hotspot, offering actionable insights for more equitable and effective conservation policies aligning with social priorities. To achieve this, we adopted the following standard theoretical frameworks and practical approaches.

**Table 2:** Multidimensional poverty indicators and their way of weighing and assessing for this study

Dimensions of poverty	Indicators	Deprived if	SDG area	Weight	Remarks
Health (1/3)	Nutrition	Any person under 70 years of age for whom there is nutritional information is undernourished.	SDG 6	1/6	–
	Child mortality	A child under 18 has died in the household in the five years before resettlement, after resettlement, and in the past five years, about 2024.	SDG 3	1/6	–
Education (1/3)	Year of schooling	No eligible household member has completed six years of schooling.	SDG 4	1/6	–
	School attendance	Any school-aged child who is not attending school up to the age at which he/she would complete grade 8.	SDG 4	1/6	–
Living standards (1/3)	Cooking fuel	Household cooking uses solid fuel, such as firewood, dung, agricultural residue, charcoal, or coal.	SDG 7	1/18	–
	Sanitation	The household has an unimproved or no sanitation facility, or it is improved but shared with other households.	SDG 6	1/18	–
	Drinking water	The household’s source of drinking water is not safe for drinking water is a 30-min or longer walking distance in a round trip from the home.	SDG 6	1/18	–
	Electricity	The household has no electricity.	SDG 7	1/18	–
	Housing	The household has inadequate housing materials in any of the three components: floor, roof, or walls.	SDG 11	1/18	–
	Assets	The household does not own more than one of these assets: radios, TV, telephone, computer, animal cart, bicycle, motorbike, or refrigerator, and does not own a car, truck, or bus.	SDG 1	1/18	–

The above-mentioned dimensions and indicators were referred to and adopted after Alkire et al. (2011). The timeline periods considered in this study, unless otherwise stated, are before resettlement, shortly after resettlement (first five years), and at present (survey year, 2024).

**Table 3:** Descriptions of the other variables beyond 10 MPI dimensions taken into consideration

Variables	Variables	Variables	Variables
1. Villages	6. Household head	11. No. of livestock holdings	16. Distance to forests
2. National Parks	7. Family members having any disability	12. No. of goats and pigs’ holdings	17. Access to forests
3. Dependency ratio	8. Do family members have any chronic diseases	13. No. of other livestock holdings	18. Religious type
4. Ethnicity	9. Distance to healthcare facilities	14. Wealth rank	19. Distance to schools
5. Size of household	10. Total land holdings	15. Distance to market	20. Social position held by family members

### Theoretical framework and practical approach

The MPI is a comprehensive measure that captures poverty through deprivations in health, education, and living standards. Developed by the united nations development programme (UNDP) and the oxford poverty and human development initiative (OPHI), MPI goes beyond non-monetary indicators to measure poverty.^19^ Initially, the poverty indices introduced by Foster and Shorrocks (1991) were generalized to the multidimensional context by Tsui^18^ and later fully reframed in 2010. This updated framework was used to assess the global MPI acute poverty in over 100 developing countries, supporting SDG 1—ending poverty in all its forms.^17^^,^^20^ It consists of three aforementioned dimensions, and tracks 10 indicators, including child malnutrition, lack of schooling, inadequate housing, and absence of basic assets, and is one of the robust approaches to tracking poverty in multidimensional aspects.^17^^,^^39^ A household is considered deprived if any child is stunted, underweight, or out of school, or if essential services like electricity, clean water, and sanitation are lacking.^17^^,^^19^ These indicators align with the human development index dimensions and weights. The detailed explanation and customization of the adopted variables/indicators of the MPI for this study are presented in Table 2.

Health dimensions include two indicators: nutrition, assessing food sufficiency year-round, and child mortality, measuring child deaths under 18 within the past five years—before, shortly after (first five years post-resettlement), and in 2024 (survey year). Similarly, the education dimension includes two key indicators: years of schooling, which evaluates whether all household members have completed at least grade 6, and school attendance, which measures whether children are currently enrolled or have left school before completing grade 8. The standard of living dimension comprises six indicators. Cooking fuel assesses the type of fuel used, such as fuelwood, dung, gas, electricity, coal, or other sources. Sanitation evaluates the availability of toilet facilities, ranging from open toileting and makeshift huts to modern sanitation systems. Drinking water considers access to water sources, including hand pumps, tap water, rivers, springs, wells, or piped water, with a requirement that the source be within a 30-min commute. Electricity is measured as a simple yes or no indicator. Housing assesses the type of dwelling, categorizing it as a hut, a mud-and-stone structure, or modern reinforced concrete buildings, either in the floor, wall, or roof types. Finally, assets are measured based on the presence of at least two of the following: TV, radio, mobile phone, telephone, bicycle, refrigerator, motorbike, scooter, single car, bus, or truck (see details in Table 2). To achieve this, we adopted the following methods for data collection and analysis in this study.

## Results

### Overall multidimensional poverty scenarios of the resettled communities

**Figure 2:**
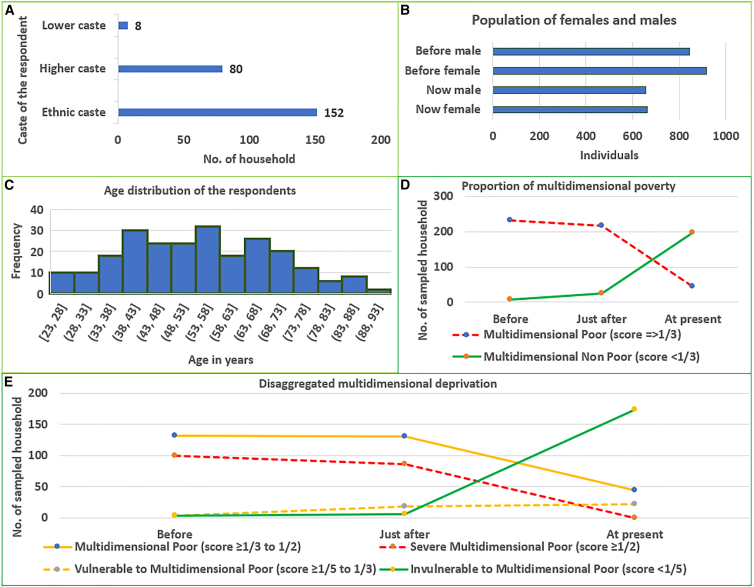
Household and respondent characteristics of the questionnaire samples The combined background information of sampled households across all study sites (five villages and two national parks) includes: (A) the distribution of ethnicity among sampled households, (B) the sex ratio before resettlement and at present, (C) age distribution of respondents, (D) proportional distribution of poor and non-poor households before, shortly after, and at present, and (E) the disaggregated status of multidimensional poverty over time. Severe multidimensional poverty declined from approximately 42% of households before resettlement to 36% shortly after, reaching 0% at present.

The overall multidimensionally poor individuals decreased from 96.7% before resettlement to 90% shortly after (in the first five years) and decreased further to 18.3% in 2024 (the survey year), while the non-poor population increased from 3.3% to 10% and then to 81.7% over the same period. Meanwhile, the percentage of severe multidimensionally poor individuals declined from 42.7% before resettlement to 36.8% shortly after and reached 0% at present, while those categorized as multidimensionally poor decreased from 56.4% to 55.6% and further to 18.8%. Meanwhile, the vulnerable population increased from 1.7% before resettlement to 7.7% shortly after and 9.4% at present, whereas the invulnerable group rose significantly from 1.7% to 2.6% and then to 74.4% during the period before resettlement, shortly after resettlement, and at present, respectively. We found that males predominantly served as household heads (85%) and constituted 65% of the interview participants, with respondents ranging in age from below 25 to above 85 years. Similarly, ethnic caste households were dominant (63.33%). Before resettlement, the female population outnumbered males, but by 2024, the sex ratio had shifted to approximately 50:50 (Figure 2).

### Multidimensional poverty analysis of the resettled community

#### Descriptive statistics of the 10 dimensions of MPI

The analysis reveals significant changes in multidimensional poverty indicators over time for households displaced from Chitwan National Park (CNP) and Parsa National Park (PNP). Health indicators showed initial improvements shortly after the intervention, particularly in child mortality, which dropped to zero in 2024. However, nutrition improvements were not sustained, with PNP households seeing a rise from 16.67% before to 21.21% in 2024. Education indicators remained relatively stable, though school attendance saw a sharp decline in the post-intervention. Living standards improved initially but deteriorated drastically by 2024, with electricity, sanitation, and housing access dropping to near zero. Cooking fuel (firewood) access saw a notable shift, with CNP households improving significantly (100%–5.56%), while PNP households had a slower decline. Asset ownership also decreases to zero in both areas by 2024 (Table 4).

**Table 4:** Observed variables’ trend of the MPI before, after, and at present in both parks in this study

Dimensions of poverty	Indicators	Before HH^a^ (%)		Shortly after HH^a^ (%)		In 2024 HH^a^ (%)	
		CNP^a^	PNP^a^	CNP^a^	PNP^a^	CNP^a^	PNP^a^
Health	Nutrition	9.26	16.67	37.04	60.61	9.26	21.21
	Child mortality	7.41	9.09	0.00	4.55	0.00	0.00
Education	Year of schooling	98.15	92.42	98.15	90.91	94.44	92.42
	School attendance	20.37	31.82	1.85	13.64	1.85	4.55
Living standards	Cooking fuel	100.00	98.48	98.15	89.39	5.56	19.70
	Sanitation	98.15	90.91	20.37	33.33	0.00	0.00
	Drinking water	20.37	0.00	87.04	0.00	0.00	0.00
	Electricity	100.00	100.00	98.15	92.42	0.00	0.00
	Housing	98.15	98.48	77.78	93.94	1.85	4.55
	Assets	27.78	51.52	18.52	31.82	0.00	0.00

a CNP refers to Chitwan National Park, PNP to Parsa National Park, and HH to the household. The timeline periods considered in this study, unless otherwise stated, are before resettlement, shortly after resettlement (first five years), and at present (survey year, 2024).

#### MPI of the respective parks

**Figure 3:**
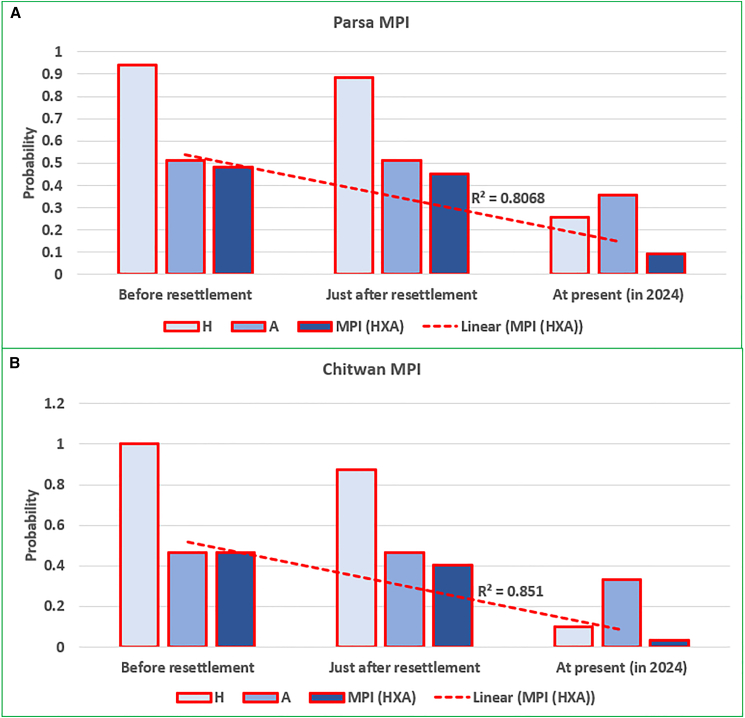
Comparison of the incidence, severity, and MPI across the parks (A) shows the MPI of resettled communities in Parsa National Park, while (B) illustrates the MPI, severity, and incidence among resettled communities in Chitwan National Park, Nepal. MPI trends indicate a significant decline (R^2^ > 0.80) among resettled communities in both parks.

Resettled communities in both (PNP and CNP) Parks have experienced significant reductions in multidimensional poverty over time (Figure 3). In PNP, the incidence of poverty (H) declined from 0.94 before resettlement to 0.26 (2024—survey year), while the intensity of poverty (A) reduced slightly from 0.51 to 0.36. Consequently, the MPI decreased from 0.48 to 0.09, reflecting substantial poverty alleviation. Similarly, in CNP, the H dropped dramatically from 1.00 before resettlement to 0.10 (2024 – survey year), and the A reduced from 0.46 to 0.33. The MPI for Chitwan resettled communities decreased sharply from 0.46 to 0.03, indicating significant improvements in living conditions. Overall, while both parks have shown notable progress, the reduction in MPI is more pronounced among Chitwan’s resettled communities, whereas poverty remains more persistent among the resettled communities in PNP, as the resettlement was only completed in 2014 and many forms of support—particularly infrastructure—are still under development. This finding suggests that, in the medium to long term, well-planned and voluntary ecological resettlement can be beneficial for both conservation objectives and poverty alleviation goals.

### Outcomes of MPI dimensions with other social-spatial variables

#### Incidence of deprivation score (H)

**Figure 4:**
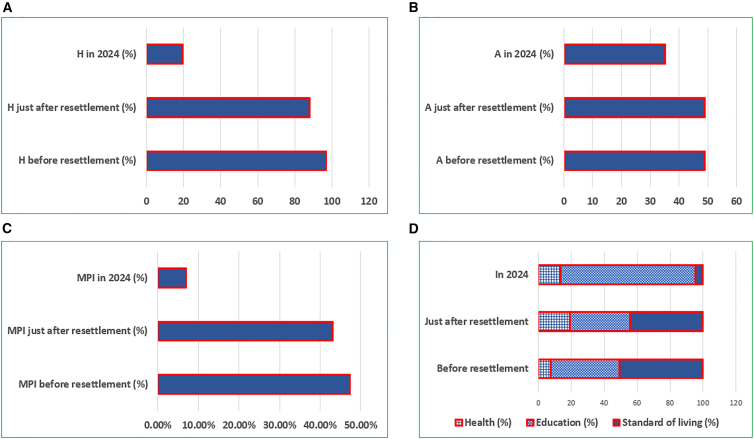
Incidence, severity, and multidimentional poverty index (MPI) of sampled households (A–D) The combined incidence, severity, and MPI of resettled communities in the study areas are presented as follows: (A) incidence of multidimensional poverty over time, (B) intensity of poverty over time, (C) overall multidimensional poverty index (MPI) trends, and (D) proportional contribution to MPI by its three equally weighted (1/3 each) dimensions—health, education, and standard of living. Unless otherwise stated, the timeline periods considered in this study are before resettlement, shortly after resettlement (first five years), and at present (survey year, 2024).

The analysis highlights a significant reduction in the H among resettled communities over time in both national parks. Before resettlement, the incidence was extremely high at 0.97. It slightly decreased to 0.88 immediately after resettlement and dropped substantially to 0.20 by 2024, indicating a marked improvement in poverty conditions post-resettlement (Figure 4A).

The statistical tests revealed significant effects of several variables on the deprivation score (H). Significant differences were found concerning village, dependency ratio, ethnicity, chronic disease, number of goats and pigs, wealth rank, religious belief, and distances to health facilities, markets, forests, and schools. In contrast, variables such as household status, disability, social status, and access to forests were not significant (Table 5).

**Table 5:** The test results of the incidence of deprivation (headcount ratio index) in relation to other independent variables among resettled communities (*n = 240*)

Independent variables	DF	Deviance	Resid. DF	Resid. Dev	F	Pr(>F)
NULL	–	–	237	10.59	–	–
Village	3	1.03	234	9.55	21.87	8.88e−12∗∗∗
Park	0	0.00	234	9.55	–	–
Dependency ratio	1	0.08	233	9.48	5.04	0.03∗
Ethnicity	2	0.20	231	9.28	6.35	0.01∗∗
Total household	1	0.02	230	9.25	1.33	0.25
Household head	1	0.01	229	9.25	0.47	0.49
Disability	1	0.01	228	9.24	0.48	0.49
Chronic disease	1	0.17	227	9.07	10.68	0.00∗∗
Distance to health	19	1.11	208	7.96	3.71	2.84e−06∗∗∗
Total land	1	0.02	207	7.95	0.96	0.33
No. of livestock	1	0.03	206	7.91	2.20	0.14
No. of goats & pigs	1	0.26	205	7.65	16.75	6.98e−05∗∗∗
Other livestock	1	0.00	204	7.64	0.19	0.66
Wealth rank	2	0.31	202	7.34	9.81	9.99e−05∗∗∗
Distance to market	16	1.70	186	5.64	6.74	2.44e−11∗∗∗
Distance to the forests	19	1.09	167	4.55	3.64	4.07e−06∗∗∗
Access to the forests	2	0.00	165	4.55	0.02	0.98
Religion belief	2	0.85	163	3.70	27.15	8.99e−11∗∗∗
Distance to school	14	1.09	149	2.60	4.96	1.68e−07∗∗∗
Social position	1	0.02	148	2.58	1.16	0.28

DF stands for Degree of Freedom, Resid. Stands for residuals, and Dev stands for deviance.

Signif. codes: ‘∗∗∗’ 0.001 ‘∗∗’ 0.01 ‘∗’ 0.05.

#### Intensity of poverty (A)

The result shows the intensity of poverty (A) among resettled communities, indicating a gradual decrease over time. Initially, the intensity was 0.49 before resettlement, remaining almost unchanged at 0.49 immediately after resettlement. By 2024, the intensity had reduced to 0.35, reflecting an overall improvement in the severity of poverty among the resettled communities (Figure 4B).

Statistical tests indicate that the intensity of poverty is significantly influenced (*p* < 0.05) by village, household size, chronic disease, distance to healthcare facilities, ownership of goats and pigs, wealth rank, distance to market, distance to forests, religious belief, and distance to school (Table 6). In contrast, ethnicity, household head (male or female), disability, total land ownership, other livestock, and access to forests were not significant.

**Table 6:** The test results of the poverty intensity index in relation to other independent variables among resettled communities (*n = 240*)

Independent variables	DF	Deviance	Resid. DF	Resid. Dev	F	Pr(>F)
NULL	–	–	237	106.454	–	–
Village	3	18.56	234	87.89	76.58	<2.2e−16∗∗∗
Parks	0	0.00	234	87.89	–	–
Dependency ratio	1	0.97	233	86.93	11.97	0.00∗∗∗
Ethnicity	2	0.21	231	86.72	1.31	0.27
Total household size	1	34.45	230	52.27	426.39	<2.2e−16∗∗∗
Household head	1	0.00	229	52.27	0.01	0.93
Disability	1	0.01	228	52.26	0.13	0.72
Chronic disease	1	1.95	227	50.31	24.11	2.37e−06∗∗∗
Distance to health	19	7.47	208	42.84	4.87	9.97e−09∗∗∗
Total land (ha)	1	0.03	207	42.81	0.40	0.53
No. of livestock	1	0.00	206	42.81	0.01	0.92
No. of goats & pigs	1	1.31	205	41.50	16.23	8.95e−05∗∗∗
Other livestock	1	0.27	204	41.22	3.39	0.07 ¥
Wealth rank	2	1.16	202	40.06	7.18	0.00∗∗
Distance to market	16	7.99	186	32.08	6.18	2.40e−10∗∗∗
Distance to the forests	19	9.00	167	23.07	5.87	8.91e−11∗∗∗
Access to the forests	2	0.16	165	22.92	0.96	0.39
Religion type	2	3.16	163	19.76	19.55	2.93e−08∗∗∗
Distance to school	14	6.38	149	13.37	5.64	1.12e−08∗∗∗
Social position	1	0.02	148	13.36	0.22	0.64

DF stands for Degree of Freedom, Resid. Stands for residuals, and Dev stands for deviance.

Signif. codes: ‘∗∗∗’ 0.001 ‘∗∗’ 0.01 ‘∗’ 0.05 ‘¥’ 0.1.

#### Household multidimensional poverty index

The results show a decreasing trend in MPI before resettlement, immediately after, and in 2024 for all resettled communities from both national parks. Before resettlement, the MPI was 47.36%, which declined to 43.03% shortly after resettlement. By 2024, it had significantly dropped to 6.87%, indicating a substantial long-term reduction in multidimensional poverty among the resettled communities (Figure 4C).

Statistical tests identify other key factors influencing household MPI. Villages, distance to market, distance to the forests, and ownership of livestock, particularly goats and pigs, significantly (*p* < 0.05) impact multidimensional poverty. Ethnicity, chronic disease, total land, religion, distance to school, and social position also show notable effects on household-level MPI across the study area (Table 7). In contrast, household status, disability, and access to forests were not statistically significant, indicating that MPI did not vary based on these variables in the study areas.

**Table 7:** Test results of the poor and non-poor household index in relation to independent variables among resettled communities (*n = 240*)

Independent variables	DF	Deviance	Resid. DF	Resid. Dev	F	Pr(>F)
NULL	–	–	237	25.44	–	–
Village	3	2.23	234	23.21	14.22	3.42e−08∗∗∗
Parks	0	0.00	234	23.21	–	–
Dependency ratio	1	0.02	233	23.19	0.37	0.54
Ethnicity	2	0.60	231	22.59	5.75	0.00∗∗
Household size	1	0.11	230	22.48	2.15	0.14
Household head	1	0.16	229	22.31	3.13	0.08 ¥
Disability	1	0.01	228	22.30	0.21	0.64
Chronic disease	1	0.44	227	21.86	8.44	0.00∗∗
Distance to health facilities	19	1.98	208	19.88	1.99	0.01∗
Total land (ha)	1	0.28	207	19.60	5.37	0.02∗
No. of livestock	1	0.86	206	18.74	16.52	7.77e−05∗∗∗
No. of goats & pigs	1	1.21	205	17.53	23.08	3.77e−06∗∗∗
Other livestock	1	0.02	204	17.51	0.43	0.51
Wealth rank	2	0.30	202	17.21	2.90	0.06 ¥
Distance to market	16	4.29	186	12.92	5.13	2.04e−08∗∗∗
Distance to the forest	19	2.81	167	10.11	2.83	0.00∗∗∗
Access to the forest	2	0.01	165	10.10	0.10	0.90
Religion belief	2	0.41	163	9.69	3.92	0.02∗
Distance to school	14	1.75	149	7.94	2.39	0.00∗∗
Social position	1	0.41	148	7.54	7.80	0.01∗∗

DF stands for Degree of Freedom, Resid. Stands for residuals, and Dev stands for deviance.

Signif. codes: ‘∗∗∗’ 0.001 ‘∗∗’ 0.01 ‘∗’ 0.05 ‘¥’ 0.1.

### Contribution of deprivation

We also outline the contribution of deprivation across health, education, and standard of living before resettlement, shortly after resettlement, and in 2024 (Table 8). Initially, the standard of living had the highest contribution (50.67%), followed by education (41.87%) and health (7.46%). After resettlement, the contributions shifted, with health increasing to 18.92%, education slightly decreasing to 36.94%, and the standard of living reduced to 44.14%. By 2024, the contribution of education dramatically rose to 81.88%, while the contributions of health and the standard of living fell to 13.41% and 4.71%, respectively, highlighting a significant shift in deprivation dynamics over time (Figure 4D; Table 8).

**Table 8:** Proportional deprivation contributions across resettled communities are assessed for three periods: before resettlement, shortly after (within five years), and the present (2024)

Period	Health (%)	Education (%)	Standard of living (%)
Before resettlement	7.46	41.87	50.67
Shortly after resettlement	18.92	36.94	44.14
In 2024	13.41	81.88	4.71

In summary, the results indicate that conservation-led displacement does not necessarily deprive resettled populations. Instead, it can help achieve dual goals: social improvement and ecological integrity. In our study areas, this was reflected in significant reductions in the MPI and improved biodiversity and environmental security, as original residential areas were restored for conservation—though this came at the cost of traditional land-based cultures. Having these results, we have highlighted some key findings for further discussion and future suggestions.

## Discussion

Balancing conservation with meeting the social needs of communities that depend on forest resources and ecosystem services is challenging. Our analysis of five ecologically resettled villages from two national parks shows that resettled communities experienced significant changes in multidimensional poverty. Similar improvement on the resettled communities’ social living conditions, production, consumption, and social integration significantly improved compared to those out-settlers in China,^20^^,^^40^ and in India.^13^ In contrast, most of the scholars reported the negative consequences on the social dimensions of the resettled communities across the global south.^5^^,^^8^^,^^14^^,^^35^^,^^41^^,^^42^ These outcomes largely reflect coercive displacement beyond voluntary resettlement, varying by space (near vs. far), time (temporary vs. permanent), and power (forced vs. voluntary), following the World Bank’s resettlement framework.^25^^,^^26^ Although they faced hardships in the first five years of post-resettlement while adapting to a new environment and materializing the resettlement policies, overall improvements were observed in health, education, and living standards, as reflected in the MPI in the medium to long term. These results suggest that poverty alleviation and conservation goals can be achieved in a win-win scenario in the long run if policies are effectively implemented with strategically planned resettlement programs. The findings affirm that conservation-led resettlement can reduce poverty while supporting ecological and environmental goals.^9^^,^^34^^,^^43^

The MPI was strongly influenced by additional factors beyond the standard 10 dimensions, including village location, service access, land and livestock ownership, and access to healthcare, education, and ecosystem services—highlighting the need to include these in assessments of forest-dependent communities in countries like Nepal. The findings suggest that combining income- and non-income-based indicators offers a more comprehensive and credible estimate of multidimensional poverty and should inform future assessments. Additionally, the MPI measures poverty beyond income by capturing deprivations in health, education, and living standards, in alignment with the human development index and a more tangible method of tracking household-level poverty.^23^^,^^39^^,^^44^ This type of analysis not only reveals poverty status but also provides insights into the incidence (H) and intensity (A) of household deprivation, helping track human development, vulnerability, and the severity of deprivation. These findings can inform decision-making across multiple dimensions, ensuring national and international social well-being targets are met without compromising ecological commitments for the benefit of both people and the planet.

### Comparative reduction of MPI of the resettled communities

Before resettlement, the MPI of the resettled communities was 47.36%, which declined to 43.03% shortly after the first five years of resettlement. By 2024, it had significantly dropped to 6.87%, indicating a substantial long-term reduction in multidimensional poverty among the resettled communities. In this period, both the incidences (H) and intensity (A) of deprivation significantly declined; in turn, MPI drastically dropped. Our findings are far better than the MPI of the national rural average (28%) and urban average (12.3%), and the national average of 17.4% in 2021, and were from 48% before resettlement,^19^^,^^21^ the sharper decline among resettled communities suggests that well-planned ecological resettlement programs,^45^ together with government’s policy focus on poverty alleviation through its all policy and programs since 2002—poverty reduction strategy paper and establishing the poverty alleviation fund (PAF)^21^^,^^22^ including monetary and land compensation schemes to the ecological resettlers, as well as free construction materials, played a crucial role.^1^^,^^46^^,^^47^ While earlier studies indicate that around 50% of Nepal’s 1995–2010 poverty reduction was due to nonagricultural income growth, remittances, and demographic shifts,^19^^,^^48^^,^^49^ the sharper declines in MPI among resettled communities highlight the long-term benefits of well-planned and voluntary ecological resettlement at the local level.

Our findings of MPI of the resettled communities are comparable with the provincial, rural, urban, and global levels. Provincially, multidimensional poverty in Nepal is highest in Karnali province (39.5%) and lowest in Bagmati province (7.0%), with other provinces showing varying rates: Koshi (15.9%), Madhesh (24.2%), Gandaki (9.6%), Lumbini (18.2%), and Sudurpashchim (25.3%).^21^ The resettled communities’ MPI is well below that of almost all provincial averages. Further, the Fourth Nepal Living Standard Survey reveals that MPI affects 12.3% of urban and 28% of rural populations, and shows that Nepal’s absolute poverty is 20.3%, which is 18.3% in urban areas and 24.7% in rural areas.^21^

We found that the percentage of severely multidimensionally poor individuals declined from 42.7% before resettlement to 36.8% shortly after and reached 0% at present in the study areas. In 2013, the human poverty index (HPI) reported that 44% of Nepalis were deprived of basic education, health, and resources.^50^ By 2019, 17.5% (5.26 million people) were multidimensionally poor, with an additional 17.8% vulnerable to poverty, an average deprivation intensity of 42.5%, and an MPI value of 0.074.^21^^,^^22^ This finding is higher than Sri Lanka (0.011) but lower than Afghanistan (0.272) and the global average for developing countries (0.088).^19^ Further, similar findings of a decrease in MPI from 0.035 in 2010 to 0.017 in 2014 were reported in China.^20^ Resettled communities often found livelihood opportunities in non-farm activities after relocating near urban centers, along national highways, and industrial centers, aligning with observations by Joshi et al. (2010). These opportunities in the locality, compounded by the progression of education, other opportunities nationally and internationally, labor contracts with several countries by Nepal, and educational migration to the first world, also contributed to downgrading the severity of poverty in the study area. Significant progress in poverty reduction during the 2000s across Nepal was first reported, primarily driven by higher remittance inflows, rapid urbanization, and increased non-farm incomes. Conducive policies also played a key role in poverty reduction. These included the poverty reduction strategy (2002–2007), the poverty reduction fund, and the declaration of open-defecation-free districts. Investments in health and hygiene, school food programs, elderly education, child enrollment initiatives, vaccination and health insurance schemes, and social security for the elderly, disabled, single women, and marginalized groups also contributed. Strengthened global partnerships to combat poverty,^9^^,^^21^ further supported these efforts. Combined with improved access and opportunities through ecological resettlement, these measures helped reduce household deprivation among resettled communities. In corroboration with our observation, a past study reported that between 1995 and 2010, around 50% of Nepal’s poverty reduction was attributed to labor income growth in nonagricultural sectors, with remittances contributing over a quarter, alongside nonfarm diversification, rising wages, and demographic shifts due to declining fertility and migration.^48^^,^^49^

### Proportional contributions on MPI by its attributes

We observed that the contribution of different deprivation attributes to Nepal’s MPI has shifted significantly over time. Before resettlement, the standard of living was the largest contributor (50.67%), followed by education (41.87%) and health (7.46%), while in 2024, education became the dominant factor (81.88%), while health and standard of Living declined to 13.41% and 4.71%. In contrast, Nepal’s 2019 MPI survey reported health, education, and standard of living contributions at 23.2%, 33.9%, and 43.0%, respectively. The sharp decline in health and standard of living contributions is linked to government policies promoting sanitation, healthcare investments, and informal education,^51^ huge investment in healthcare and informal education (adult literacy),^52^^,^^53^ along with improved household assets, energy sources, drinking water, and housing conditions.^54^^,^^55^ Meanwhile, education’s rising MPI share reflects widespread incomplete schooling, early dropout rates, and a shift toward unskilled labor and foreign employment.^21^^,^^55^ These trends illustrate evolving poverty dynamics and the changing challenges in addressing multidimensional poverty in resettlement contexts.

### Associated additional attributes in determining MPI

Results show that several additional attributes of the social and spatial dimensions, other than the 10 dimensions of the MPI initially recommended, have a strong association with MPI in our dataset. These additional significant attributes out of 20 under consideration in our study were villages, distance to market, to forests, to school, to health care facilities, ownership of the size of land, livestock, including goats and pigs, ethnicity, family members having chronic diseases, type of religion, and social position, found to differentially impact the multidimensional deprivation. These attributes are both income-based and non-income-based and strongly influence the 10 attributes of the 3 dimensions of MPI (education, health, and standards of living). Similar observations of additional factors were recommended in MPI estimation, in alignment with the human development index and the monitoring purpose^32^ as an improvement on the existing dimensions, consisting of various disciplines.^16^^,^^39^ As such, the existing 10 factors of MPI are not absolute but relative to the other factors. For instance, the type of housing directly depends on the household income source and the land availability.^32^ Similarly, enrollment of the children in the school largely depends on the accessibility of the education facilities and distance to, and access to the health care and food access directly influence the distance to the corresponding facilities and the market and/or the forest services (wild food such as mushrooms, wild yams, and other fruits).^56^^,^^57^ Therefore, the absolute estimation of MPI dimensions relying only on 10 factors may not reflect the accurate estimation of the multidimensional deprivation score at the household level in forest-resource-dependent communities, like in the case of forest dwellers who have been resettled for conservation reasons. Therefore, as suggested by Alkire and Shen (2017), our findings also affirm that including both income-based and non-income-based indicators provides a more reliable and robust estimation for tracking poverty at the household level and informing eradication efforts.

### Scope and limitation of the study

This study contributes to understanding ecological resettlement by using the MPI to track poverty dynamics across multiple dimensions—health, education, and living standards—before, immediately after, and years’ post-resettlement (2024). By focusing on these broader poverty indicators, the research offers a comprehensive view of the socio-economic impacts on affected communities, challenging assumptions about long-term impoverishment. However, the study’s scope is limited to a specific group of resettled communities and cannot be directly compared with non-resettled communities. Additionally, biases in the available survey data and sampling weights may affect the generalizability of the results. Future research could explore longitudinal studies, comparative analyses across regions, and qualitative approaches to deepen the understanding of both resettled and stationary communities’ experiences. Additionally, future analyses should incorporate multiple social factors such as loss of livelihoods, household displacement, gender-disaggregated analysis, cultural erosion, and psychological or traumatic distress, along with external influences like government policies, socio-spatial dynamics, and global economic shifts. This would help expand the scope of the MPI and improve sustainable planning by aligning human development tracking with poverty indicators without compromising conservation goals.

Nevertheless, this study’s findings have important implications for global conservation policies and sustainable development strategies, highlighting the need to balance environmental conservation with human development goals.^9^^,^^34^^,^^43^ As we observed, if ecological resettlement improves poverty indicators in the long term, it could guide future programs with better planning and support. Conversely, enduring impoverishment would emphasize the need for stronger social safeguards and inclusive strategies. This research contributes to the global discourse on sustainable development, ensuring conservation efforts are equitable and aligned with the sustainable development goals (SDGs) and beyond in combating poverty elimination and improving human development indicators.^9^^,^^43^ Moreover, findings offer insights into assessing poverty among displaced populations globally, highlighting the need to revise MPI indicators to include both income- and non-income-based measures for their broader relevance and credibility. Furthermore, the results provide valuable insights for informing policies and conservation strategies involving human displacement and tracking multidimensional poverty alongside the human development index at various spatial and temporal scales.

In conclusion, global poverty reduction efforts began with the Earth summit, which encouraged integration of poverty alleviation into national policies. Since 2000, frameworks like the millennium development goals (MDGs) and sustainable development goals (SDGs) have prioritized this issue, though balancing conservation and social needs remains challenging. Our analysis of five ecologically resettled villages from two national parks shows significant reductions in multidimensional poverty. Despite initial hardships adapting to new environments, overall improvements were observed in health, education, and living standards, as reflected in the MPI. These findings challenge the assumption that conservation-led displacement inevitably results in lasting impoverishment. Further, results indicate that conservation-led displacement can achieve both social improvement and ecological integrity. In our study areas, it reduced multidimensional poverty and enhanced biodiversity, though it came at the cost of traditional land-based cultures. Findings suggest that when strategically planned ecological resettlement programs are effectively implemented, policies for achieving social welfare and conservation goals can be harmonized in a win-win scenario.

Moreover, the MPI showed strong associations with factors beyond its standard 10 dimensions. Variables such as village location, ethnicity, land and livestock ownership, and access to markets, forests, schools, and healthcare should be included in assessments of poverty of the forest-dependent communities to improve the accuracy, credibility, and broader applicability of MPI beyond poverty tracking to human development monitoring. Furthermore, local wealth-ranking practices also proved useful in identifying deprivation. While MPI effectively captures deprivations in health, education, and living standards, it overlooks critical factors such as loss of traditional livelihoods, cultural erosion, social fragmentation, and psychological distress. Our findings suggest that combining income- and non-income-based indicators offers a more realistic assessment of multidimensional poverty. Future studies should incorporate these social and external dimensions and extend across broader spatial and temporal scales to evaluate the long-term sustainability of resettlement impacts. These insights support the need for resettlement strategies that balance ecological goals with human well-being, aligning with national, and global development targets.

Moving forward, to strengthen the effectiveness of ecological resettlement, first, governments should develop integrated resettlement policies that ensure long-term livelihood opportunities, access to basic services, and compensation packages aligned with local contexts. Second, expand the MPI framework by incorporating locally relevant income and non-income factors, such as access to markets, education, health, land ownership, and cultural dislocation to improve poverty assessments in forest-dependent, gender, and displaced communities. Third, promote targeted investments in education to address its rising contribution to poverty, especially in resettled areas where incomplete schooling and early dropout rates remain high. Fourth, ensure community-centered planning that respects local cultural protocols, strengthens trust, and enables meaningful participation through resettlement design, monitoring, and benefit-sharing. Finally, future research should focus on longitudinal and comparative studies that examine the social, psychological, and cultural impacts of displacement across diverse ecological and governance contexts to guide equitable and sustainable development practices.

## Resource availability

### Lead contact

Further information and request for resources and clarification should be directed to an will be fulfilled by the lead contact, Hari Prasad Pandey (hari.pandey@usq.edu.au, pandeyhp123@gmail.com).

### Materials availability

This study did not generate new, unique reagents.

### Data and code availability

• Data: Data used in this study cannot share due to ethical reason of the Human Research Ethics Committee [Ethics application ETH2023-0568 (HREC)] at the University of Southern Queensland, Australia, and the official permission of the Government of Nepal, Ministry of Forests and Environment, Department of National Parks and Wildlife Conservation (DNPWC) [Permission letter number: 2080/08-Eco-214; Correspondence number: 3020]. In other words, due to ethical considerations in human research, the data cannot be shared publicly to maintain confidentiality. However, prior informed consent was obtained from all the participants for their volunteer participation.
• Code: This paper does not report original code.
• Other items: Any additional information and clarification required regarding this paper can be reached up to the lead contact point upon a valid and reasonable request.

## Acknowledgments

The first author sincerely thanks the Australian Government and the University of Southern Queensland for the Research Training Program Stipend Scholarship and the International Fees Research Scholarship, which made this study possible. The first author also acknowledges the Government of Nepal for granting study leave for this research. The authors extend their gratitude to the parks and forest officials, security personnel across the Terai Arc Landscape (TAL) Area of Nepal, 10.13039/501100022422WWF Nepal for support in data collection, the TAL Program, the Zoological Society of London (ZSL) Nepal Office, the National Trust for Nature Conservation Nepal, and the local communities for their insightful opinions and cooperation during fieldwork. Special thanks to field research assistants Manisha Poudel, Aakankshya Shrestha, Chintamani Panjiyar, and Prem Bahadur Bhujel for their assistance with data collection. However, this research did not receive any specific funding.

## Author contributions

H.P.P., conceptualization, data curation, methodology, visualization, and writing the original drafts; A.A., review and editing, supervision, and english proofing; T.N.M., review & editing, supervision, and discussion. All authors review and approve the manuscript.

## Declaration of interests

The authors declare that there are no financial or other conflicts of interest among the authors or supporting organizations related to this study, the data used, or any other aspects of this research.

## STAR★Methods

### Method details

#### Study area

The study was conducted across all resettlement sites of two national parks: Chitwan National Park (NP) and Parsa NP (Figure 1), both situated within the Terai Arc Landscape (TAL). These parks were once home to forest-dependent, impoverished, and indigenous communities whose livelihoods were intricately linked to natural resources.^11^^,^^58^^,^^59^ Resettlement has further exacerbated their vulnerability by detaching them from these essential resources, disrupting their traditional way of life.^45^

Historically, these national parks have played a significant role in biodiversity conservation, particularly for species such as tigers, one-horned rhinoceroses, and Asiatic elephants, while also protecting the region’s unique ecosystems.^58^ Chitwan National Park (CNP) was designated a UNESCO World Heritage Site in 1984, and Parsa National Park (PNP), located adjacent to it in the east. This contributes to a contiguous landscape with interconnected forest habitats to support a wide range of floras, fauna, and ecosystems at all levels (genetic, species, and ecosystems) of biodiversity conservation. Additionally, these parks, especially their buffer zones, are vital for preserving the cultural heritage of local and indigenous communities, forming a socio-ecological landscape that exemplifies the deep interconnection between nature and culture.^58^^,^^60^ These NPs, as one of the oldest national parks, have had significant incidences of ecological resettlement in the past,^1^^,^^45^ becomes an ideal site for fulfilling the objectives by providing testimony and a crucial avenue for referencing to understand the MPI in conservation-induced resettlements. Insights from these parks can serve as a valuable reference for global efforts to balance social needs with conservation goals.

#### Sampling design

Based on an extensive review of the literature, identification of potential sites, and discussions with experts and government authorities, we employed a multi-stage sampling approach within a participatory research framework to collect primary data. In the first stage, we selected protected areas where resettlement occurred for the establishment or expansion of protected areas. In the second stage, we prioritized protected areas of international importance (e.g., World Heritage Sites) with transboundary significance. In the third stage, resettled villages were identified. Finally, households were randomly selected from the list of all resettled households at the time of resettlement, ensuring a minimum sampling intensity in each village (see details in Table 1). From this series of stages, we finally came to select the Chitwan National Park and Parsa National Parks for this study. These two parks were selected from Nepal’s 12 national parks for their socio-economic and ecological importance and the pressures they face from development and the displacement of forest-dependent Indigenous communities to address conservation goals and community needs.^58^

For a comprehensive overview of the resettlements, we included all resettlement sites from these two national parks, totaling five villages (Table 1). We then employed a random sampling technique to select resettled households within the selected villages for data collection, minimizing bias and ensuring equal representation. To further reduce potential bias, if a selected household declined or was unavailable, we randomly selected alternative households from the sampling frame to obtain the required level of sampling intensity by referring to similar studies.^61^^,^^62^

#### Data collection and categorization

Ethical approval for this research was granted by the Human Research Ethics Committee at the University of Southern Queensland, Australia (Ethics Application ETH2023-0568 HREC). Official authorization was also obtained from Nepal’s Ministry of Forests and Environment and the Department of National Parks and Wildlife Conservation, in compliance with national regulations. This study on ecological resettlement and poverty alleviation in the Terai Arc Landscape (TAL) of Nepal strictly adhered to ethical standards, including anonymity, informed consent, the right to refuse responses, and respect for local cultural protocols. Personal identifiers were removed to protect participants’ confidentiality and ensure data integrity. Informed consent was obtained after clearly explaining the study’s purpose, procedures, and potential implications in locally understood terms. Local cultural norms were respected by engaging community leaders, following traditional protocols, and using native languages to build trust and ensure ethical, culturally sensitive data collection. Household lists of resettled families were acquired from the Chitwan and Parsa National Park Offices, and households were randomly selected for interviews. Participants were fully informed about the voluntary nature of the study, their right to withdraw at any time, and the confidentiality of their responses, in accordance with the ethical guidelines of the authors’ institutions. The sample questionnaire is provided in the Supplementary file (S1).

Data was collected using three main methods: semi-structured questionnaire interviews (*SSQI = 240*), focus group discussions (*FGD = 5*), and key informant interviews (*KII = 25*). These methods were complemented by field observations, formal and informal discussions, transect walks, and participation in conservation committee meetings and workshops. For SSQI, we targeted a sampling intensity of at least 10%, which is a standard recommendation in social studies to ensure accurate representation,^63^^,^^64^ particularly in relatively homogeneous communities like those involved in ecological resettlement (ER), as in our study.

Each response is assigned a deprivation score based on household-level deprivations in MPI indicators. The health and education indicators are weighed at 1/6 each, while each of the six living standards indicators is weighed at 1/18 (see details in Table 2). A person’s deprivation score is the weighted sum of their deprivations, ranging from 0 to 1, with higher values indicating greater poverty. MPI complements monetary poverty measures by identifying who is poor, their deprivation profiles, and the severity of their poverty, highlighting interconnected non-monetary deprivations. A household is considered multidimensionally poor if its total deprivation score is 1/3 (33.33%) or higher. Scores between 1/5 and 1/3 (20–33.33%) indicate vulnerability to poverty, while scores of 1/2 (≥50%) or higher reflect severe poverty.^17^^,^^19^^,^^44^

#### Data analysis

##### MPI estimation and contribution

Background information was analyzed using descriptive statistics, whereas structured data was analyzed in an inferential manner. The MPI has been estimated in two steps, i.e., estimating the incidences of poverty (H) and the intensity of deprivations (A).^20^^,^^39^^,^^44^ The headcount ratio, *H*, or incidence of multidimensional poverty, is the proportion of multidimensionally poor people in the population:

H=q/n

Where *q* is the number of multidimensionally poor people in the population, and *n* is the total population. The intensity of poverty, *A*, reflects the average proportion of the weighted component indicators in which multidimensionally poor people are deprived. For multidimensionally poor people, only those with a deprivation score *s*_*i*_ greater than or equal to 33.3%, their deprivation scores are summed and divided by the total number of multidimensionally poor people:

$$A=\left(\text{Sum}\;\text{of}\;s_{i}\;\text{of}\;\text{all}\;\text{population}\;\text{from}\;1\;\text{to}\;q\right)/q$$

Where *s*_*i*_ is the deprivation score that the _*i*_th multidimensionally poor person experiences. The deprivation score *s*_*i*_*,* of the _*i*_th multidimensionally poor person can be expressed as the sum of the weights associated with each indicator *j* (*j* = 1, 2, …, 10) in which person *i* is deprived.

$$s_{i\;}=c_{i1}+c_{i2}+\ldots +c_{i10}$$

Where *c*_*j*_ is the weight associated with indicator *j* (either 1/6 or 1/18), and the weights sum to 1. The MPI value is the product of two measures: the incidence of multidimensional poverty and the intensity of poverty^44^:

MPI=H×A

The contribution of dimension *d* to multidimensional poverty can be expressed as follows:

$${\text{Contrib}}_{d}=\left(\text{sum}\;of_{\;j}\;to_{\;d}\times \text{sum}\;\text{of}\;1\;\text{to}\;q\;\text{of}\;c_{\text{ij}}\;/\;n\right)/\;\text{MPI}$$

Where _d_ is health, or education, or standard of living. All the parameters defined above are estimated using the sampled data and weights according to the rule of sampling theory.^63^^,^^64^

##### Statistical tests

We developed a generalized linear regression model, followed by three specific models, each corresponding to a dependent variable (H, A, and MPI), to be tested against 20 independent variables (Table 3).

The variables were analyzed using a Generalized Linear Model (GLM) with a quasi-Poisson distribution and a logit link function, after testing various linear, general, and mixed-effect models to determine the best fit for the data and to minimize deviance and error.^65^ Further, we selected the quasi-Poisson model for final analysis because our data showed evidence of overdispersion, which violates the assumptions of a standard Poisson model.^66^^,^^67^ While the negative binomial model is a common remedy for overdispersion in ecological research,^68^ our comparative diagnostics indicated that the quasi-Poisson model offered superior goodness-of-fit and more stable estimates for our dataset.

The generic model is as follows:

(Model 1) $$\log\left(Y_{\text{ij}}\right)=f\;\left(b_{\text{ij}}+\beta_{\text{ij}}X_{\text{ij}\;}+e_{\text{ij}}\right)$$

Where *Y*_*ij*_ refers to the dependent (responses) variables (H, A, and MPI); *f* is the function (a Quasi-Poisson distribution with logit function); and *b*_*ij*_ refers to the constants of the tested models; *β*_*ij*_ refers to the estimated coefficient for independent (predictors) variables; *X*_*ij*_ refers to the independent variables as defined above; and *e*_*ij*_ is the residual of deviance of each model. The customized models would look as follows (Model 1.1, 1.2, and 1.3).

(Model 1.1) $$H=b_{H}+\beta_{1}X_{1\;}+\beta_{2}X_{2\;}+\ldots +\beta_{20}X_{20}\cdots$$

(Model 1.2) $$A=b_{A}+\beta_{1}X_{1\;}+\beta_{2}X_{2\;}+\ldots +\beta_{20}X_{20}\cdots$$

(Model 1.3) $$\text{MPI}=b_{\text{MPI}\;}+\beta_{1}X_{1\;}+\beta_{2}X_{2\;}+\ldots +\beta_{20}X_{20}\cdots$$

Where *H, A*, and *MPI* are the mean incidence of deprivation, the intensity of poverty, and the multidimensional poverty index, respectively; *b*_*H*_*, b*_*A*_*,* and *b*_*MPI*_ are the constants of the tested respective models; *β*_*1,*_ *β*_*2 ….*_ *Β*_*20*_ are the coefficients of the respective variables, and *X*_*1*_*, X*_*2*_*, … X*_*20*_ are independent variables other than MPI predefined indicators as specified in Table 3, taken into consideration for this study. The visual presentation of the observed relationship is presented as simply as possible using bar graphs and pie diagrams to ease interpretation and communication. Descriptive analyses were carried out in the MS Excel platform, referring to the technical note of the global multidimensional poverty index, 2021.^44^ Additionally, inferential analyses, including Chi-square tests and modeling, were performed in RStudio using various libraries.^69^
