# Supplementary material for: Integrating social welfare and conservation: Improvements in multidimensional poverty index outcomes from ecological resettlements in Nepal
Source: iScience. 2026 Apr 3;29(5):115580. doi: 10.1016/j.isci.2026.115580 (PMC13122836; doi:10.1016/j.isci.2026.115580)
Supplement: Document S1. Table S1 [file mmc1.pdf]

iScience, Volume *29*

Supplemental information

Integrating social welfare and conservation: Improvements in multidimensional poverty index outcomes from ecological resettlements in Nepal

Hari Prasad Pandey, Armando Apan, and Tek Narayan Maraseni

Supplementary File

Table S1: Sample checklist for Multidimensional Poverty Indicators assessment and their way of measurement considered for this study.

Dimensions of poverty	Indicators	Questions	Before resettlement	After resettlement	In 2024 (survey year)
Health	Nutrition	Have/had you gotten sufficient food for your family? If not, how many months?	[ ] yes, [ ] no, …………	[ ] yes, [ ] no, ………	[ ] yes, [ ] no, ………
	Child mortality	Did you lose your children under 18 years of age in the past 5 years? If yes, how many?	[ ] yes, [ ] no ……..	[ ] yes, [ ] no …….	[ ] yes, [ ] no …….
Education	Year of schooling	Have/had every member of your family completed grade 6? How many did so?	[ ] yes, [ ] no ……….	[ ] yes, [ ] no ………	[ ] yes, [ ] no ………
	School attendance	Have/had your children not attended or left the school before grade 8? How many did so?	[ ] yes, [ ] no …….	[ ] yes, [ ] no …..	[ ] yes, [ ] no …..
Living standards	Cooking fuel	What was/is the cooking fuel in your household? (dung, fuelwood, …, electricity)	…………	………….	………….
	Sanitation	What type of toilet did/do you have? (open, hut, modern)	…………	………	………
	Drinking water	What was/is your source of water? How far it was/is? <30 minutes, 30+)	……….., ………	………., ……..	………., ……..
	Electricity	Did/do you have electricity in your house?	[ ] yes, [ ] no	[ ] yes, [ ] no	[ ]yes, [ ] no
	Housing	Had/have you had adequate floor, roof, or walls of the house? If not, which one?	[ ] yes, [ ] no, ……….	[ ] yes, [ ] no …………..	[ ]yes, [ ] no …………..
	Assets	Did/do you own assets like radios, TVs, phones, computers, …, or a car/bus/truck?	[ ] yes, [ ] no ……….	[ ] yes, [ ] no ……..	[ ]yes, [ ] no ……..
